# Breastfeeding and prevention of childhood obesity: a narrative review of behavioral, hormonal, and microbiome programming

**DOI:** 10.3389/fnut.2026.1800487

**Published:** 2026-05-29

**Authors:** Noor Abu Dheir, Hadia Radwan, Haydar Hasan, Dimitrios Papandreou, Falak Zeb, Dana N. Abdelrahim, May Aly Abdelaziz, Nagham Nihad Abdoh, Maysm N. Mohamad, Ayesha S. Al Dhaheri, Lily Stojanovska, Leila Cheikh Ismail

**Affiliations:** 1 Department of Clinical Nutrition and Dietetics, College of Health Sciences, University of Sharjah, Sharjah, United Arab Emirates; 2 Department of Nutrition and Health, College of Medicine and Health Sciences, United Arab Emirates University, Al Ain, United Arab Emirates; 3 Institute for Health and Sport, Victoria University, Melbourne, VIC, Australia; 4 Nuffield Department of Women’s & Reproductive Health, University of Oxford, Oxford, United Kingdom

**Keywords:** breastfeeding, childhood obesity, early-life nutrition, gut microbiome, human milk bioactive, leptin, metabolic programming

## Abstract

Childhood obesity has emerged as a major public health concern worldwide, with increasing prevalence in high-income countries. Growing evidence suggests that early-life nutrition, particularly breastfeeding, plays a critical role in reducing obesity that shaping long-term metabolic health. This narrative review highlight association between breastfeeding duration, exclusivity, and childhood obesity risk, synthesizing current evidence while exploring potential biological and behavioral mechanisms. Most studies report that breastfeeding, particularly when exclusive and sustained for longer durations, is associated with a reduced risk of childhood overweight and obesity. Evidence consistently shows that breastfed infants exhibit healthier growth trajectories, lower rates of rapid weight gain, and reduced adiposity compared to formula-fed infants. Several studies also identify plausible biological mechanisms, including appetite regulation, favorable insulin responses, and the influence of bioactive components in human milk, such as leptin, adiponectin, ghrelin, insulin-like growth factors (IGFs) and gut microbiome modulating factors. Nonetheless, some studies show weak or non-significant associations, often attributable to methodological differences, inconsistent breastfeeding practices, or inadequate adjustment for confounders such as maternal BMI and socioeconomic status. Current literature indicates that breastfeeding may serve as a protective factor against childhood obesity, highlighting its relevance in early-life nutrition and long-term health outcomes. This review underscores that promoting exclusive breastfeeding for at least 6 months should be a cornerstone of public health strategies to prevent childhood obesity.

## 1 Introduction

Childhood overweight and obesity are major global public health concerns because they are strongly associated with adverse health outcomes in adulthood, including non-communicable diseases (NCDs). Their prevalence has increased steadily in both high-income and low and middle-income countries, raising serious concerns for future population health (1). Early childhood obesity is a strong predictor of obesity in adulthood (2). Obesity in children not only increases the risk of cardiometabolic and respiratory diseases but is also linked with low self-esteem, social discrimination, and emotional difficulties (3).

Childhood obesity stems from various factors, such as genetic influences, individual behaviors like physical activity, sleep patterns, and television habits, as well as dietary choices and their interplay (4). Many researchers have explored several methods for preventing obesity among children, and one potential intervention that has been suggested is promoting breastfeeding as a preventive measure against childhood obesity (4). Epidemiological studies indicated that breastfeeding is linked to a decreased risk of overweight and obesity in children ages 2–12 (5). The World Health Organization (WHO) recommends exclusively nursing infants during the initial 6 months of age (6). Ensuring optimal nutrition during the critical 1,000-day period from conception to a child’s second year of age sets the stage for an excellent start in life, yielding long-term advantages. The brain, adipose tissue, endocrine, and immunological systems all expand quickly during this window, making them more vulnerable to environmental and nutritional programming. Nutritional exposures at this time can have long-lasting effects on energy balance, insulin sensitivity, gut microbiota composition, and appetite regulation, which can shape the risk of obesity and metabolic diseases later in life (7).

Given the complex interplay of genetics, lifestyle, and diet in obesity development, early-life interventions such as breastfeeding have been proposed to mitigate long-term obesity risk. Breastfeeding has been linked to lower obesity rates and other health benefits for both child and mother. Exclusive breastfeeding can influence DNA methylation and gene expression related to metabolism and the immune system, potentially reducing the risk of obesity, metabolic disorders, and other conditions later in life (8). Human breastmilk contains diverse substances with potential mechanistic roles in metabolic health during early childhood, including macronutrients, micronutrients, metabolic hormones, adipokines, miRNAs, and inflammatory markers (9, 10). Moreover, breastfeeding can regulate infant metabolism through a variety of mechanisms including growth factors, immune factors, microbiota, and appetite hormones (11, 12). Breastfeeding shapes the gut microbiota in early life, both directly by exposure of the neonate to the milk microbiota and indirectly, via maternal milk factors that affect bacterial growth and metabolism such as human milk oligosaccharides (HMOs), secretory IgA, and anti-microbial factors (13). The potential of breastmilk to modulate the offspring’s early gut microbiota is a promising tool for obesity prevention.

**Figure 1:**
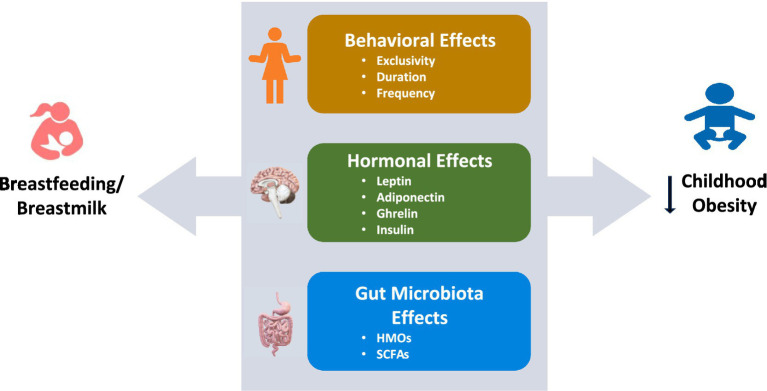
Diagram shows a summary of breastfeeding targets that leads to the prevention of childhood obesity.

This narrative review synthesizes current evidence on the relationship between breastfeeding practices (breastfeeding duration, exclusivity, and breast milk composition) and childhood obesity. To date, a clear protective effect of breastfeeding on prevention of childhood obesity by targeting metabolic, hormonal and microbiome pathways has not been demonstrated. Therefore, we aim to clarify how early-life breastfeeding practices influence obesity risk and provide insights to inform effective interventions and public health policies and to offer an updated and integrative perspective on the relationship between breastfeeding and childhood obesity by bridging epidemiological evidence with underlying biological mechanisms. Furthermore, by examining how breastfeeding programming behavioral, hormonal, and gut microbiome regulation that reduce the risk of adiposity and metabolic abnormalities summarized in Figure 1. Through this comprehensive approach, the review contributes a novel and timely understanding of how breastfeeding functions as a modifiable early-life factor in reducing the risk of childhood obesity.

## 2 Methodology

This narrative review was informed by a structured literature search of electronic databases, including PubMed, Scopus, and Web of Science, to identify studies examining the relationship between breastfeeding and childhood overweight, obesity, or related metabolic outcomes. We searched for articles published from January 2000 to March 2025 using combinations of the following keywords and MeSH terms: “breastfeeding,” “human milk,” “childhood obesity,” “overweight,” “metabolic programming,” “gut microbiome,” “adipokines,” “leptin,” “adiponectin,” and “early-life nutrition.” Studies were included if they: (1) involved human participants; (2) assessed breastfeeding exposure (duration, exclusivity, or breastmilk composition) in relation to anthropometric, metabolic, hormonal, or microbiome outcomes in infants, children, or adolescents; and (3) were original research articles (cohort, case–control, cross-sectional studies, randomized trials) or systematic reviews/meta-analyses. We excluded case reports, animal-only studies, conference abstracts without full text, and articles not published in English. Reference lists of key articles and recent reviews were also screened to identify additional relevant studies.

## 3 Epidemiological evidence associating breastfeeding practices and childhood obesity

Breastfeeding can be a key factor and first line of defense against childhood obesity. A case–control study demonstrated that an association between breastfeeding and a lower risk of overweight and obesity in 12–18-year-olds, showing a dose-dependent effect according to the breastfeeding duration (14). Similarly, a large cohort investigated Scottish three-year-old children, found that breastfeeding was associated with a reduced risk of obesity (15). Breastfeeding has been linked with a lower incidence of obesity among children (16), and the degree of protection increasing if breastfeeding is continued (17). Specifically, breastfeeding for a duration of 13–25 weeks was associated with a 38% reduction in obesity risk, whereas breastfeeding for more than 26 weeks was associated with a 51% reduction (18).

One study reported that exclusive breastfeeding reduced obesity risk and body fat in children aged 9–11 years compared to those who were never breastfed (4). Another meta-analysis of 25 studies demonstrated a dose–response relationship between breastfeeding duration and reduced risk of childhood obesity, indicated that longer exclusive breastfeeding is linked to greater protection (17). The idea that longer breastfeeding duration and exclusivity are linked to healthier weight trajectories in children is supported by data from both large-scale cohort studies and meta-analyses. The evidence for a causative role is strengthened by the dose–response association shown in several studies, especially when exclusive breastfeeding lasts longer than 4–6 months (19). An analysis of nationally representative data from the United States (NHANES, 2009–2020) involving 3,211 children aged 2 to <7 years (weighted ≈10 million) showed that short-term breastfeeding up to 6 months was associated with lower odds of obesity in certain subgroups. Protective associations were particularly evident among children of mothers aged ≥35 years and among preschoolers aged 3–4 years, with odds ratios as low as 0.15 in some comparisons; furthermore, later introduction of formula or other milks appeared to strengthen the preventive effect (19). In a cross-sectional study of over 9,000 children aged 5–6, found that breastfed children had a lower prevalence of obesity than formula-fed peers, with a dose–response relationship between breastfeeding duration and obesity risk (20). Similarly, cohort studies following nearly 9,000 children (7,000 girls and boys) aged 9–14 reported that longer breastfeeding duration was associated with reduced risk of overweight compared to formula-fed infants (21). Armstrong and Reilly also found that breastfeeding reduced obesity prevalence in 32,000 Scottish children aged 3–4 (15). Horta et al.’s systematic review and meta-analysis further confirmed that breastfeeding was associated with a 13% reduction in overweight/obesity and potentially lowers the risk of type II diabetes mellitus (22). Moreover, exclusive breastfeeding was linked to a lower likelihood of overweight at 18 months, while breastfeeding for up to 6 months and introducing solid foods after 6 months were associated with reduced odds of infant overweight at 12 months (23). Furthermore, breastfeeding has been shown to have positive effects on maternal weight control. Mothers who breastfeed their infants tend to lose weight more rapidly at postpartum and have a lower risk of long-term weight retention compared to those who do not breastfeed (24).

Taken together, these cohort studies and meta-analyses consistently support a modest but meaningful protective association between breastfeeding and childhood overweight or obesity, with stronger effects observed for longer durations and higher exclusivity of breastfeeding. At the same time, the magnitude of this association varies across settings and study designs, and is partly attenuated when maternal and socioeconomic confounders are rigorously adjusted, underscoring the need to interpret breastfeeding as one important component within a broader constellation of obesity risk factors rather than a standalone solution.

Some cohorts report no significant associations between breastfeeding duration and later obesity after rigorous adjustment for confounders such as maternal education, smoking, and BMI (25). Socioeconomic and cultural factors also strongly influence both breastfeeding practices and obesity risk. For example, higher-income and more educated mothers are more likely to breastfeed and have environments conducive to healthier childhood behaviors. Additionally, maternal obesity influences not only breastfeeding duration but also the hormonal composition of breast milk, potentially attenuating the metabolic benefits of breastfeeding for obesity prevention (26). In a similar vein, Palaska et al. found that the frequency of overweight, particularly in boys, was negatively correlated with prolonged exclusive breastfeeding in a cross-sectional Greek cohort of children aged 2–5. Nonetheless, maternal BMI continued to be a reliable indicator of child weight, highlighting the interaction of environmental and genetic factors in families (27). Unlike bottle-feeding, breastfeeding mothers cannot directly monitor the exact volume of milk their infant consumes. Consequently, they rely on infant cues such as satiety signals, which encourage trust in the child’s ability to self-regulate intake rather than promoting overconsumption (28).

These discrepant findings likely reflect differences in how breastfeeding is measured, the length of follow-up, and the extent to which studies can account for confounding by maternal health, household environment, and socioeconomic conditions. Therefore, breastfeeding should be interpreted as one important early-life factor within a broader risk profile rather than as a standalone explanation for later obesity risk.

## 4 Breastfeeding targeted mechanisms

The protective effects of breastfeeding against childhood obesity arise from multiple, interconnected biological and behavioral mechanisms. First, breast milk shapes the infant gut microbiome by promoting the growth of beneficial bacteria such as Bifidobacterium species, which support healthy metabolism, enhance intestinal maturation, and reduce inflammation, factors associated with lower obesity risk. Second, breastfeeding supports better appetite and energy intake regulation through hormonal regulation. Unlike formula feeding, breastfeeding allows infants to self-regulate their milk intake, reducing the likelihood of overfeeding and promoting healthier growth patterns during infancy. Third, human milk contains bioactive components such as leptin, insulin, ghrelin, adiponectin, HMOs, resistin, obestatin, insulin-like growth factor-1, copeptin, apelin, and nesfatin, which play important roles in appetite control, adipocyte differentiation, and metabolic programming. These hormones may activate anorexigenic or orexigenic pathways depending on energy stores and needs (29). Additionally, early exposure to the flavors presents in breast milk which vary with maternal diet may shape taste preferences and encourage healthier eating behaviors later in childhood, indirectly reducing the risk of excessive weight gain (4).

## 5 Breastfeeding practices and childhood obesity

Several variables may contribute to breastfeeding’s protective effect against obesity progression (30).

Behavioral differences in feeding patterns may partly explain the protective association between breastfeeding and later obesity. Breastfed infants typically feed more frequently and regulate the volume of milk they consume, which can reduce the risk of overfeeding and rapid early weight gain, whereas formula feeding often involves more parental control over volume and feeding schedules (28, 31, 32). In addition, exposure to maternal diet-derived flavors in breast milk may shape later food preferences and facilitate acceptance of healthier complementary foods, providing another pathway through which breastfeeding influences long-term dietary behaviors (33, 34).

During lactation, differences between breastfeeding and formula-feeding affect maternal–infant interactions and feeding practices, which in turn may influence infants’ ability to self-regulate intake and growth. These early differences can contribute to long-term variations in energy balance and food intake regulation between breastfed and formula-fed infants (28). A recent data from the 2-year Mother Infant Study Cohort (MISC), which followed 256 women from the third trimester of pregnancy to 18 months postpartum in Sharjah, Dubai, and Ajman, indicate that maternal factors such as Arab nationality, pre-pregnancy overweight or obesity, higher gestational weight gain, and lower physical activity are associated with an increased risk of infants being overweight at 6, 12, and 18 months (23).

Parents who choose to breastfeed often follow healthier lifestyles, which may influence their children’s nutrition and activity patterns. Nutrient composition is another key factor. Infant formulas generally have higher energy density and protein content than human milk, which may stimulate insulin and IGF-I release, accelerate growth, and increase fat deposition, contributing to earlier adiposity rebound and obesity risk (35–37). Differences in fat quality are also important. Formulas with a higher omega-6 to omega-3 ratio may promote inflammation and adipocyte development, while omega-3 fatty acids in breast milk support appetite regulation and reduce obesity risk (38). Growth trajectories also differ, as breastfed infants initially lose more weight and regain it more slowly than formula-fed infants (39). Growth rates converge in the first 3 months, but thereafter, formula-fed infants show greater weight gain (40). This slower pattern in breastfed infants may reduce fat accumulation and protect against later obesity (41). Finally, early nutritional exposures can influence gene expression and metabolic programming, linking breast milk composition during pregnancy and lactation to lifelong disease risk (28). Together, these mechanisms highlight how breastfeeding promotes healthier growth patterns and lowers obesity risk (42).

## 6 Hormonal components of breastmilk and metabolic programming

**Figure 2:**
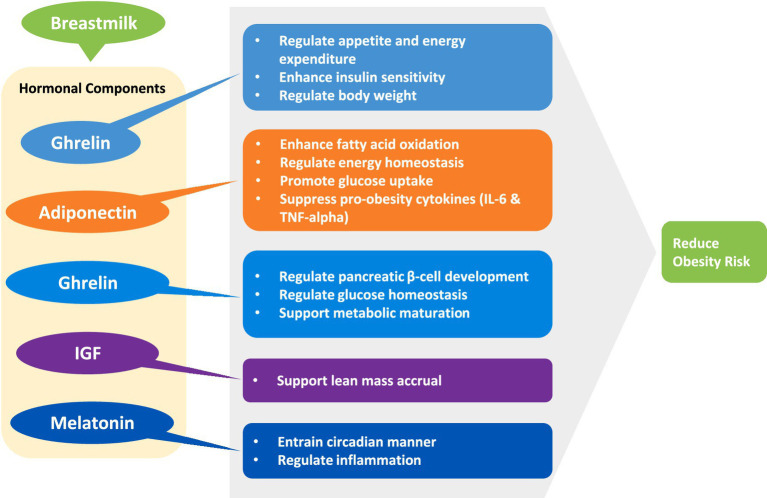
The metabolic targets of breastmilk hormones.

Breast milk is a biologically active fluid that provides optimal nutrition along with key bioactive compounds that influence child metabolism, appetite control, energy balance, fat accumulation, hormonal regulation and body composition. Figure 2 summarizes the metabolic targets of hormonal components in breastmilk that reduce the risk of childhood obesity. Among these, leptin and adiponectin play central roles in metabolic programming. Leptin supports appetite regulation and energy expenditure by acting on the hypothalamus to reduce food intake and promote satiety. Adiponectin, enhances insulin sensitivity, promotes fatty-acid oxidation, and supports healthier adipocyte function. Together, these hormones help regulate energy balance, reduce early fat accumulation, and may lower the long-term risk of childhood obesity (33, 34).

Moreover, growth patterns and adiposity rebound may be impacted by the variations in protein composition between human milk and infant formula (43, 44). Higher leptin levels have been observed in whole milk compared to skimmed milk, likely due to its association with fat droplets or fat-bound proteins (45). Building on these findings, leptin present in breast milk may also transfer into the infant’s bloodstream, potentially influencing early appetite regulation. This is supported by evidence that leptin receptors are located in the stomach’s epithelial cells and absorptive cells of the small intestine in both mice and humans (46).

Breastfeeding may alter gene–environment interactions that affect metabolic health in addition to regulating hormones. According to Danaie et al., long-term nursing reduces genetic susceptibilities to obesity by activating epigenetic pathways that involve insulin sensitivity and leptin. The notion that breast milk bioactives affect gene expression and metabolic programming in addition to acting hormonally is supported by this new evidence (47). Extending these experimental insights, studies in rats have shown that those receiving physiological doses of oral leptin during the nursing period exhibited lower body weight and adiposity in adulthood, enhanced insulin sensitivity, and a reduced preference for high-fat foods compared to controls when later exposed to either chow or high-fat diets (48). These findings suggest that breast milk leptin may not only act as a satiety signal and short-term regulator of food intake in newborns but could also exert long-term programming effects on energy balance, body weight regulation, and metabolic health (49, 50).

The unique combination of breastmilk nutrients and bioactive compounds such as adiponectin, and (HMOs) has been consistently associated with a lower risk of childhood obesity (51). Adiponectin circulates in biologically active high-molecular-weight forms and is known to enhance insulin sensitivity and fatty acid oxidation while being inversely associated with adiposity in adults and children, suggesting its role in energy homeostasis and metabolic health (52). Studies have demonstrated that higher concentrations of adiponectin in human milk are associated with lower infant weight-for-age and weight-for-length z-scores during early infancy, indicating an influence on early growth trajectories that may help mitigate rapid adipose accumulation, a known predictor of later obesity risk (53). The molecular mechanisms underlying this effect are believed to include enhanced insulin sensitivity via adiponectin receptor signaling pathways (AdipoR1/R2) that activate AMP-activated protein kinase (AMPK), thereby promoting fatty acid oxidation and glucose uptake in peripheral tissues, as well as anti-inflammatory actions that suppress pro-obesity cytokines such as TNF-α and IL-6 (54). Additionally, adiponectin may interact with other hormonal and growth factors in breast milk to modulate adipogenesis and appetite regulation during critical windows of metabolic programming in infancy. Adiponectin, exhibiting antihyperglycemic, antiatherogenic, and anti-inflammatory properties, could have important clinical benefits in terms of development of therapies for the prevention and/or for the treatment of obesity and obesity-related diseases (55). In liver, adiponectin activates glucose transport and inhibits gluconeogenesis via AMPK, whereas adiponectin activates fatty acid oxidation and decreases inflammation through the PPARα pathway (56). In addition, adiponectin, in liver, enhances insulin sensitivity promoting phosphorylation of the insulin receptor and of the adaptor protein insulin receptor substrate 1 (IRS-1) (57). In adipose tissues, adiponectin increases basal glucose uptake and enhances insulin-stimulated glucose uptake through AMPK activation (56).

In addition to leptin and adiponectin, other breast milk components may contribute to its protective role against obesity by influencing energy regulation and fat metabolism in infants. These components contain ghrelin, insulin, insulin-like growth factors (IGF-1 and IGF-2), and resistin, each influencing growth and metabolic processes. The IGF axis supports lean mass accrual and longitudinal growth; however, evidence linking milk IGF levels to later obesity risk remains limited and inconsistent.

It was also reported that active ghrelin concentration correlates positively with infant growth rate and weight gain (58). Ghrelin, an orexigenic hormone, may support metabolic maturation and hypothalamic development, while milk-derived insulin may help regulate pancreatic β-cell development and glucose homeostasis during infancy (59). Thus, ghrelin content in breastmilk may be a factor through which breastfeeding could influence infant feeding behavior and body composition (60). Insulin is present in breastmilk, and its primary source is maternal blood but there is evidence that mammary epithelial cells can also produce insulin. Its concentration is highest in the colostrum and then decreases to levels detected in maternal serum (61). Studies show that the intermediate levels of insulin in breast milk are associated with lower infant weight and lean mass. However, higher levels might be negatively associated with body weight (10, 62). Scientists found that melatonin in breastmilk is associated with improved sleep duration and efficiency. The intake of melatonin by newborns helps to keep their physiological mechanisms in a circadian manner. Moreover, melatonin is a powerful antioxidant involved in regulating inflammation. It can also reestablish the balance of the gastrointestinal microbiota and significantly improve metabolic disturbances (10). Therefore, it is one of the most important ingredients of breastmilk as it plays a role not only in the circadian rhythm of the infant, but it may also affect immunity and the incidence of obesity later in life. This highlights how breast milk functions as a natural protective factor against obesity through its diverse biological properties.

## 7 Breastmilk improves gut microbiome and immunometabolic programming

**Figure 3:**
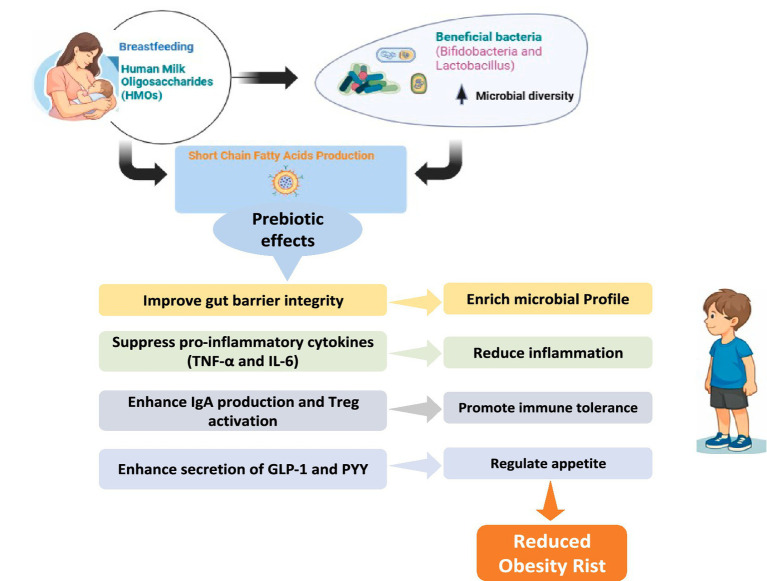
Breastfeeding entrains gut microbiota and its immunometabolic programming, summarizing key pathways linking human milk, the infant microbiome, and immunometabolic regulation as described in Victora et al. (78) and Kiely et al. (72).

Breastfeeding plays a crucial role in programming the infant gut microbiome and shaping immunometabolic regulation during early life, through a combination of bioactive nutrients, immune factors, and microbiota-modulating components (Figure 3). HMOs selectively promote the growth of Bifidobacterium and other commensals, leading to a gut microbial profile enriched in taxa associated with improved barrier integrity, lower inflammation, and healthier metabolic outcomes (63, 64). This microbiota influences immune development by driving regulatory T cell differentiation, enhancing mucosal IgA production, and reducing pro-inflammatory cytokine signaling, thereby promoting immune tolerance and reducing systemic low-grade inflammation implicated in metabolic dysfunction (65).

The immunometabolic benefits of breastfeeding are further supported by recent mechanistic research. Neonatal immune cell signaling pathways are shaped by human breast feeding, according to single-cell transcriptome analysis (66), whereas a preprint describes new effects of breastfeeding on an infant’s metabolism (67). Further research demonstrates benefits for gastrointestinal health (68), and intestine gluconeogenesis, a crucial metabolic route for energy regulation, is improved by breastfeeding (69).

Breast milk influences metabolic programming not only through endocrine signaling but also via gut microbiome shaping, largely driven by HMOs. HMOs act as prebiotics promoting Bifidobacteria-dominant microbiota, leading to production of short-chain fatty acids (SCFAs) such as butyrate and propionate. SCFAs contribute to metabolic regulation by, (a) enhancing glucagon-like peptide-1 (GLP-1) and peptide YY (PYY) secretion (appetite suppression), (b) improving insulin sensitivity, (c) reducing systemic inflammation via Treg activation. These gut-mediated immune-metabolic effects help explain why breastfed infants tend to have lower adiposity and reduced rapid weight gain, key predictors of childhood obesity (70). This protective effect is partly driven by HMOs, which selectively promote Bifidobacterium growth, contributing to a distinct microbiome in breastfed infants (71). HMOs are pivotal bioactive components in HBM, fourth most abundant after water, lactose, and lipids. HMOs show significant resilience against enzymatic digestion within the gastrointestinal tract. This quality allows them to reach the far end of the gastrointestinal tract in a mostly unchanged state, where resident bacteria can metabolize them (72). They play a role in regulating neonatal immunity by influencing the responses of both host epithelial and immune cells within the newborn’s gut. Such colonization may reduce obesity risk, as these sugars reach the infant distal intestine intact, thereby serving as a fermentable substrate for specific intestinal microbes, including Firmicutes, Proteobacteria, and especially infant-associated Bifidobacterium spp. which help to shape the infant gut microbiome. Bacteria utilizing HMOs are equipped with genes associated with their degradation and a number of carbohydrate-active enzymes known as glycoside hydrolase enzymes have been identified in the infant gut (72). Alterations in early gut microbiota are closely linked to long-term health, influencing immune and endocrine systems and increasing susceptibility to conditions such as obesity and allergies (73). Early excessive weight gain in infancy, for example, has been associated with obesity at age three (74), consistent with findings that microbiome patterns in infancy can predict obesity risk years later (75).

Breast milk also contains antimicrobial peptides (e.g., lactoferrin), cytokines, and immunoglobulins that shape microbial colonization patterns, while microbial fermentation of HMOs yields short-chain fatty acids (SCFAs) such as acetate, propionate, and butyrate which act as signaling molecules regulating host energy metabolism, glucose homeostasis, and adiposity through pathways including GPR41/43, AMPK activation, and modulation of enteroendocrine hormones such as GLP-1 and PYY (76, 77). Collectively, these interactions help explain why breastfed infants exhibit reduced risk of obesity, improved insulin sensitivity, and more favorable inflammatory profiles later in life compared to formula-fed peers, supporting the concept that breastfeeding induces immunometabolic programming via microbiome-immune-metabolic crosstalk during critical developmental windows (78). Additionally, it supports the establishment of a beneficial gut microbiome, which further contributes to metabolic regulation and a reduced obesity risk (33, 34). Breast milk harbors a diverse microbiota, including Staphylococcus, Streptococcus, and lactic acid bacteria, whose composition is influenced by delivery mode, gestational age, maternal health, BMI, and pregnancy weight gain (42, 79, 80). Notably, obese mothers produce milk with reduced microbial diversity, characterized by higher Staphylococcus and lower Bifidobacterium counts compared to mothers of normal weight (79, 81). Bifidobacterium spp. are enriched in the gut of breastfed and healthy infants; their presence may offer protection against later disease risk (82, 83). Supporting this, a study found that children maintaining normal weight at age seven had higher infant Bifidobacteria counts, whereas those who became overweight showed elevated *Staphylococcus aureus* levels during infancy (75).

Maternal obesity further shapes infant gut colonization, with babies born to obese mothers showing distinct bacterial patterns compared to those of lean mothers, including differences in Blautia, Eubacterium, Oscillibacter, and Faecalibacterium spp. (84, 85). Socioeconomic status also modulates these differences, suggesting that both biological and environmental factors jointly influence the infant microbiome and its role in obesity risk (85).

## 8 Limitations

This review has several limitations. First, most of the available evidence is observational, which limits the ability to infer causality and leaves residual confounding by factors such as maternal BMI, education, socioeconomic status, and health behaviors. Second, heterogeneity in definitions of breastfeeding (duration, exclusivity, timing of complementary feeding), outcome measures, and follow-up periods complicates direct comparison across studies and may contribute to inconsistent findings. Third, publication bias and the predominance of studies from high-income settings may limit the generalizability of conclusions to low- and middle-income countries and marginalized populations. Finally, as a narrative rather than a formal systematic review, our search strategy may not have captured every eligible study, although we attempted to minimize this by screening reference lists of key articles and recent reviews.

## 9 Conclusion

Breastfeeding emerges from the available evidence as an important protective factor against the development of childhood overweight and obesity. Consistent findings across observational studies and meta-analyses indicate that both breastfeeding initiation and longer duration are associated with healthier weight trajectories in childhood. However, the strength of this association may be influenced by maternal and environmental factors, including maternal body mass index, gestational weight gain, socioeconomic conditions, workplace support, and cultural practices. Breast milk contains bioactive components, such as leptin, adiponectin, and other metabolic hormones, as well as immunological and microbial factors, that may influence appetite regulation, energy balance, gut microbiome development, and metabolic programming during critical early-life windows. These mechanisms highlight breastfeeding as a complex biological process with potential long-term implications for metabolic health. Future research should focus on clarifying these mechanisms using well-designed longitudinal studies, while also examining genetic and epigenetic factors that may modify the relationship between breastfeeding and obesity risk. In parallel, public health efforts should prioritize the development and implementation of comprehensive policies and interventions that support breastfeeding, including breastfeeding-friendly workplace policies, community-based support programs, and education for healthcare providers. Strengthening structural supports—such as paid maternity leave, workplace lactation rooms, trained lactation counselors in primary care, and full implementation of the World Health Organization (WHO) and/or United Nations International Children’s Emergency Fund (UNICEF) Baby-Friendly Hospital Initiative—is essential to optimize breastfeeding practices and promote long-term health benefits for both children and mothers.
